# The Role of Endothelial Progenitor Cells (EPCs) and Circulating Endothelial Cells (CECs) as Early Biomarkers of Endothelial Dysfunction in Children with Newly Diagnosed Type 1 Diabetes

**DOI:** 10.3390/cells14141095

**Published:** 2025-07-17

**Authors:** Milena Jamiołkowska-Sztabkowska, Sebastian Ciężki, Aleksandra Starosz, Kamil Grubczak, Marcin Moniuszko, Artur Bossowski, Barbara Głowińska-Olszewska

**Affiliations:** 1 Department of Pediatrics, Endocrinology, Diabetology with Cardiology Division, Medical University of Bialystok, 15-274 Bialystok, Poland; 2 Clinical Department of Internal Medicine, Endocrinology and Diabetology, National Medical Institute of the Ministry of Interior and Administration, 02-507 Warsaw, Poland; 3 Department of Regenerative Medicine and Immune Regulation, Medical University of Bialystok, 15-089 Bialystok, Poland; 4 Department of Allergology and Internal Medicine, Medical University of Bialystok, 15-089 Bialystok, Poland

**Keywords:** type 1 diabetes, children, endothelial function, vascular complications

## Abstract

The aim of this study is to assess endothelial progenitor cells (EPCs) and circulating endothelial cells (CECs) at the time of type 1 diabetes (T1D) recognition concerning patients’ clinical state, remaining insulin secretion, and further partial remission (PR) occurrence. We recruited 45 children that were admitted to hospital due to newly diagnosed T1D (median age 10.8 yrs), and 20 healthy peers as a control group. EPC and CEC levels were measured at disease onset in PBMC isolated from whole peripheral blood with the use of flow cytometry. Clinical data regarding patients’ condition, C-peptide secretion, and further PR prevalence were analyzed. T1D-diagnosed patients presented higher EPC levels than the control group (*p* = 0.026), while no statistical differences in CEC levels and EPC/CEC ratio were observed. Considering only T1D patients, those with better clinical conditions presented lower EPCs (*p* = 0.021) and lower EPC/CEC ratios (*p* = 0.0002). Patients with C-peptide secretion within a normal range at disease onset presented lower EPC/CEC ratios (*p* = 0.027). Higher levels of EPCs were observed more frequently in patients with higher glucose, decreased fasting C-peptide, and lower stimulated C-peptide (all *p* < 0.05). The presence of DKA was related to higher EPC/CEC ratios (*p* = 0.034). Significantly higher levels of CECs were observed in patients who presented partial remission of the disease at 6 months after diagnosis (*p* = 0.03) only. In the study group, positive correlations of CECs with age, BMI at onset, and BMI in following years were observed. EPC/CEC ratios correlated positively with glucose levels at hospital admission and negatively with age, BMI, pH, and stimulated C-peptide level. We reveal a new potential for the application of EPCs and CECs as biomarkers, reflecting both endothelial injury and reconstruction processes in children with T1D. There is a need for further research in order to reduce cardiovascular risk in children with T1D.

## 1. Introduction

The fact that individuals affected by type 1 diabetes (T1D) are predisposed to significantly elevated risks of mortality, primarily from cardiovascular diseases (CVD) [1], is widely acknowledged. It has been proven that even young people with T1D exhibit subclinical vascular damage [2]. Endothelial abnormalities of both small and large blood vessels induced by diabetes are already present even before the onset of overt clinical microvascular or macrovascular complications, which may indicate that endothelial dysfunction serves as an early indicator of cardiovascular risk [3]. Diabetes, by promoting oxidative stress, chronic inflammation, and reduced nitric oxide production, contributes to impaired endothelial function [4,5]. Furthermore, chronic hyperglycemia and glucose variability are recognized as being key contributors to endothelial dysfunction in individuals with diabetes [6,7,8]. Ongoing research in this area has provided evidence that people with long-lasting type 1 diabetes exhibit a decrease in circulating endothelial progenitor cells (EPCs) compared to those who experience a shorter duration of the disease [9]. Moreover, patients with diabetes type 1 present increased circulating endothelial cell (CEC) levels than healthy individuals [10]. This is a significant finding, as EPCs are produced in bone marrow and contribute to neovascularization and the rebuilding of a damaged endothelium, and their reduced number and activity is proven to be connected to greater incidence of both micro- and macrovascular complications of diabetes [8]. Under exposure to ischemia or hypoxia, hypoxia-induced factor 1 (HIF-1) is activated, which, in turn, enhances the production and release of stromal-derived factor 1 (SDF-1)—a crucial factor that mobilizes EPCs to repair damaged the endothelium [11]. It has been previously demonstrated that diabetes, regardless of its type, significantly reduces the recruitment of EPCs from bone marrow into peripheral circulation, impairs their function, and increases apoptosis of these cells [12].

On the other hand, CECs (cells of the exfoliated endothelium) are secreted into the bloodstream during vascular injury; thus, they can serve as a marker of ongoing endothelial damage [13,14].

The characteristics of the above-mentioned cells and endothelium function at type 1 diabetes diagnosis are currently poorly described, especially in the youngest group of newly diagnosed patients. These patients have the greatest potential to minimize the risk of long-term complications, as long as actions aimed at achieving optimal glycemic control are introduced in the initial phase of the disease [15]. It has been demonstrated that early and effective glycemic control among T1D individuals is associated with the longer preservation of pancreatic beta cell function and, consequently, higher C-peptide levels [16]. Noteworthy, C-peptide serves not only as a marker of endogenous insulin production, but also acts as an active biological agent with antioxidant, anti-apoptotic, and anti-inflammatory properties [17]. Research indicates that patients diagnosed with type 1 diabetes who have detectable C-peptide levels tend to develop fewer microvascular complications than those without, potentially due to reduced endothelial injury [18]. This association could be explained by the protective effects of C-peptide on the endothelium [18,19]. The early stage of diabetes diagnosis appears to be a critical point in the disease course. This highlights the need to maintain optimal glycemic control and prolong the duration of partial remission for as long as possible, as this decreases the risk of subsequent diabetes-related complications, as well as enables us to identify biomarkers that can predict the risk of negative vascular outcomes later in life.

Taking the above factors into consideration, we evaluated EPC and CEC levels at T1D diagnosis considering patients’ clinical conditions, residual insulin secretion, and the occurrence of partial remission. Our aim was to evaluate EPCs and CECs’ potential as indicators of early endothelial injury and predictors of vascular consequences in the future. We also attempted to identify the mechanisms underlying the observed relationships that may also play a role in developing novel therapies designed to lower cardiovascular risk in T1D patients.

## 2. Materials and Methods

We recruited 45 children who were just diagnosed with T1D in the Department of Pediatrics, Endocrinology, Diabetology, and Cardiology Division (Medical University of Bialystok, Poland). All of the patients then stayed under the routine care of the outpatient clinic for the following 24 months. These children were recognized as having T1D according to ISPAD diagnostic criteria recommendations [20]. Our patients were of Caucasian origin. The median age of our patients was 11 years (from 6 to 18 yrs). Children younger than 5 years with diabetes diagnosed using T1D immunological/genetical screening, those who lacked satisfactory control visit compliance, or those who were transferred to another medical center during observation were excluded from this study. We analyzed anthropometric data like weight, height, BMI, BMI–standard deviation score (BMI-SDS), and gender. HbA1c and total daily insulin requirement (DIR) [U/kg/24 h] were assessed at the day of discharge from hospital (diabetes onset), after 3 and 6 months, and at the 1st and 2nd year follow-up to determinate partial remission (PR) occurrence (defined as HbA1c  <  53 mmol/mol [<7%] and DIR  <  0.5 U/kg/24 h) [21]. Patients achieving an insulin demand < 0.5 U/kg/24 g with normoglycemia before the hospital discharge, but still with significantly elevated HbA1c found at disease onset, were also qualified as entering PR. C-peptide levels were assessed at illness onset and after 2 years of insulin therapy to determine the residual endogenous insulin secretion (normal range: 1.1–4.4 ng/mL, reported by the laboratory).

For the purpose of this study, in further analyses comparing EPC and CEC levels at different clinical situations, patients were divided into groups regarding the presence of diabetic ketoacidosis (DKA) at disease recognition (defined as capillary blood pH < 7.30 and/or bicarbonate <18 accompanying hyperglycemia, altogether with ketonemia/ketonuria) and clinical condition at admission (Table 1).

In total, 20 children, admitted to the department due to reported chest pain, weakness, or mild arrythmias, but who were otherwise healthy, were recruited as a control group. The full clinical characteristics of the study groups are presented in Table 1.

The protocol of this study was approved by the Ethical Committee at the Medical University of Bialystok (R-I-002/345/2015). Prior to blood sample collection, written informed consent was obtained from the participants’ parents/legal guardians and from the participants themselves if they were aged >13 years.

Venous blood samples were collected for laboratory analyses. In patients with T1D, samples were collected 3–5 days after subcutaneous insulin therapy was initiated after recovery from DKA. C-peptide levels were assessed at illness onset (fasting and 2 h meal-stimulated) and after 2 years (fasting) with the use of the electrochemiluminescence method (ECLIA) (detection limit assessed as 0.01 ng/mL for the assay). HbA1c was determined using high-performance liquid chromatography.

**Figure 1:**
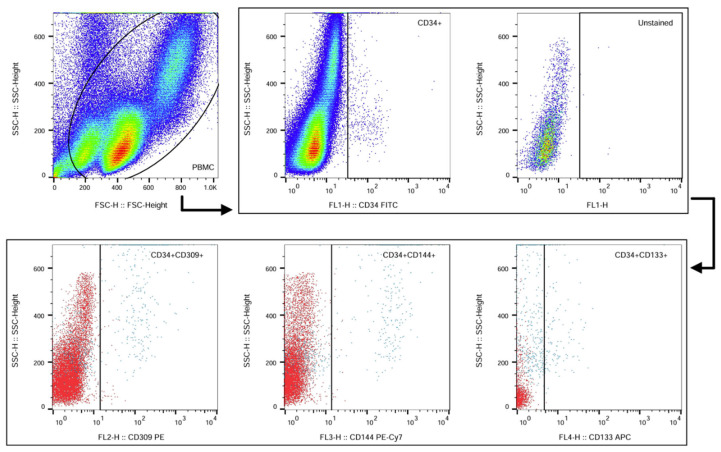
Gating strategy of endothelial progenitors and circulating cells. Selection of CD34+ cells within cells, with the morphology of mononuclear cells based on FSC and SSC properties. Subsequent gates set on CD309+ and CD133+ (for EPCs) or CD144+ (for CECs) using Boolean gating were implemented. All necessary fluorescence minus one (FMO) (red) or unstained controls are demonstrated.

EDTA-anticoagulated peripheral blood was used. Peripheral blood mononuclear cells (PBMCs) were isolated with the use of density gradient (Pancoll 1.077 g/L; PAN Biotech, Aidenbach, Germany) and centrifugation. Obtained PBMCs were washed in phosphate-buffered saline (PBS with no Ca^2+^ and Mg^2+^; Corning, Manassas, VA, USA) and preserved in liquid nitrogen with a cryoprotectant: fetal bovine serum (FBS; PAN Biotech, Aidenbach, Germany) with 10% DMSO (Sigma-Aldrich, Darmstadt, Germany). Following the thawing procedure, PBMCs’ viability was evaluated microscopically using 0.4% trypan blue solution and hemacytometer of the Bürker chamber. Flow cytometric immunostaining was performed on 1 × 10^6^ cells, with the following fluorochrome-conjugated monoclonal antibodies: anti-CD34 FITC (clone 581), anti-CD133 APC (clone W6B3C1), anti-CD144 PE-Cy7 (clone55-7H1), and anti-CD309 PE (clone 89106) (BD Biosciences, Sand Diego, CA, USA). Incubation of samples for 25 min (at room temperature in the dark), was followed by centrifugation with PBS at 400 g for 5 min. Stained cells were fixated (CellFix; BD Biosciences, Erembodegen, Belgium) and stored at 4 °C until acquisition using a FACS Calibur flow cytometer (BD Biosciences, San Jose, CA, USA). For batch variability control, the flow cytometer’s settings were standardized across all runs (daily maintenance, data acquisition times, samples volume, and cell content), with samples stained and processed using the same protocols (in context of the antibodies’ lot, time, temperature, and buffer conditions). Data processing was performed with the use of FlowJo software version 10.7.2 (TreeStarInc., Ashland, SA, USA). Endothelial-related progenitor and circulating cells were initially gated on the basis of forward- and side-scatter (FSC/SSC) properties to exclude debris and enrich for mononuclear cells. Subsequent gating on CD34-positive cells was implemented as a marker for vascular-associated progenitor cells. Boolean gating was applied within CD34+ to distinguish selected cell populations on the basis of the co-expression of CD309 (VEGFR-2) and CD133 (prominin-1; progenitor cells marker) (EPCs—endothelial progenitor cells) or the presence of CD144+ (vascular endothelial cadherin) (CECs—circulating endothelial cells) (gating strategy presented graphically within Figure 1). The selected markers used and the gating strategy were validated previously and based on our experiences with EPC and CEC populations [22]. Established cell subsets are presented in terms of their frequency within PBMCs, and with the absolute events reported. According to the obtained data, we decided to divide patients with T1D into subgroups regarding the median levels of the studied cell populations.

Statistical analyses were performed using Statistica 14.1 (StatSoft, Krakow, Poland). Continuous variables were tested using the Kolmogorov–Smirnov test with Lilliefors correction and Shapiro–Wilk tests for normal distribution. Most of the studied parameters were not normally distributed; therefore, descriptive statistics were calculated as medians with the interquartile range: median (IQR). To compare continuous variables, Mann–Whitney U and ANOVA Kruskall–Wallis tests were used, and the χ^2^ test with Yates correction was used to compare categorical variables between groups. Correlations between the studied variables were assessed using Spearman correlation coefficients. A multiple linear regression analysis was performed to detect independent determinants of EPC and CEC levels and of EPC/CEC ratios. *p* values < 0.05 were considered statistically significant. Considering the sample size, for Mann–Whitney U tests with assumed α = 0.05, an effect size of 0.7, and an allocation ratio of 2.25, to obtain the power of the test (1-β) at 0.8, a total sample size of 64 would be needed. For the χ^2^ test with assumed α = 0.05 and an effect size of 0.4, to obtain the power of the test at 0.8, a total sample size of 69 would be needed.

## 3. Results

A total number of 45 T1D patients (aged 6–18 years) and 20 healthy peers (aged 9–17.5 years) were recruited in this study. The characteristics of the study participants are presented in Table 2.

**Figure 2:**
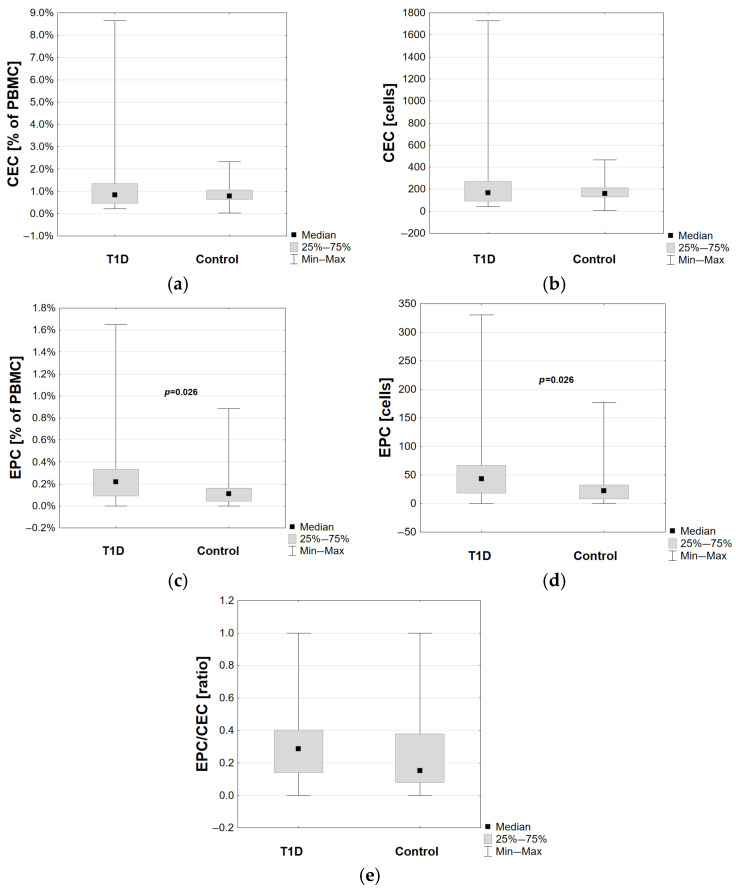
Comparison of (**a**,**b**) CECs—circulating endothelial cells, (**c**,**d**) EPCs—endothelial progenitor cells, and (**e**) EPC/CEC ratio between T1D patients and the control group. Statistically significant *p* values are presented. (T1D—type 1 diabetes, Control—control group).

Regarding the studied cells, T1D patients presented higher EPC levels than the control group (*p* = 0.026), while there was no statistical difference in CEC levels and EPC/CEC ratio, as shown in Figure 2.

**Figure 3:**
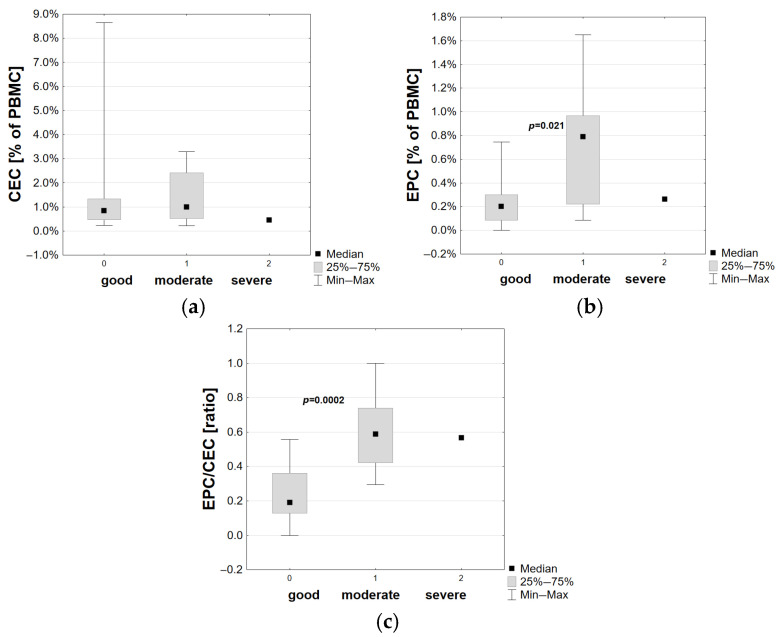
Comparison of (**a**) CECs—circulating endothelial cells, (**b**) EPCs—endothelial progenitor cells, and (**c**) EPC/CEC ratio depending on T1D patients’ clinical conditions at the disease onset. Statistically significant *p* values are presented.

Considering only T1D patients, children with better clinical conditions presented lower EPCs (*p* = 0.021) and lower EPC/CEC ratios (*p* = 0.0002), as shown in Figure 3. Patients with C-peptide secretion within a normal range at disease onset also presented lower EPC/CEC ratios (*p* = 0.027).

An analysis of the clinical features potentially affecting levels of EPCs, CECs, and EPC/CEC ratios revealed that EPC levels > median were observed more frequently in patients with higher glucose (*p* = 0.025) and lower stimulated C-peptide (*p* = 0.033) at diagnosis, while EPC levels < median were observed more frequently in patients with C-peptide level within a normal range (*p* = 0.017). Higher EPC/CEC ratios were more frequent among those presenting DKA at diagnosis (*p* = 0.034). In relation to PR occurrence, the presence of CEC levels > median was significantly more prevalent in patients who presented PR at 6 months after diagnosis (*p* = 0.03); see Table 3.

As shown in Table 4, in the study group, positive correlations of CECs with age, BMI at onset, and BMI in the following years were observed. EPC/CEC ratios correlated positively with glucose level at hospital admission and negatively with age, BMI, and pH and stimulated C-peptide levels.

A linear regression analysis showed that pH was the main factor determining EPC/CEC ratio (β= −0.059, *p* = 0.002). In the logistic regression analyses, clinical condition at hospital admission determined the EPC/CEC ratios (*p* < 0.00001) and age, and stimulated C-peptide (>median) determined CEC levels > median (both *p* < 0.00001).

## 4. Discussion

The aim of our research was to evaluate the utility of EPCs and CECs as biomarkers of endothelial dysfunction at T1D onset (in the context of a patient’s clinical condition and partial remission occurrence) and to attempt to find the mechanisms responsible for the reported associations. In contrast with the majority of the available research, which analyzed the levels of these cells at a latter phase of diabetes duration, our study focuses on their evaluation at the time of diagnosis. This will facilitate a better understanding of early endothelial alterations in the course of type 1 diabetes.

**Figure 4:**
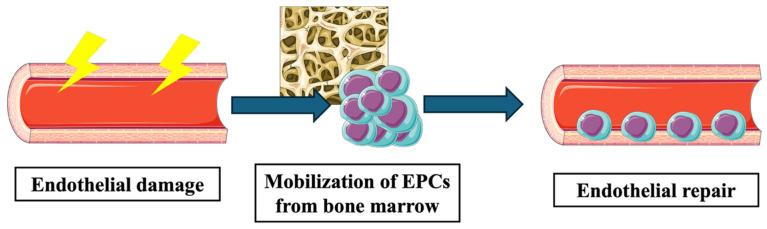
Representation of the mechanism by which elevated levels of EPCs may serve as a potential early indicator of endothelial dysfunction in individuals with type 1 diabetes.

We discovered that children with newly diagnosed T1D exhibit higher EPC levels compared to healthy controls. This observation supports earlier findings, including our own [9,23]. This may reflect an early compensatory response to subclinical endothelial damage already present at the initial stages of the disease; we hypothesize that multiple endothelial-damaging factors lead to enhanced recruitment of EPCs from bone marrow. Although it is established that long-term diabetes is associated with the diminished levels and functionality of EPCs, leading to reduced endothelial repair capacity [23], it seems that, in the early phase of diabetes, their potential is preserved. Thus, elevated EPC levels among T1D individuals with short disease durations may serve as an early marker of endothelial injury. A schematic overview of the potential link between elevated EPC levels and early endothelial dysfunction in T1D is provided in Figure 4.

On the other hand, some studies reported reduced EPC numbers in T1D children compared to healthy individuals, yet they presented a longer disease course and poor metabolic control [10]. This may suggest that EPC dynamics are influenced by disease progression and glycemic status.

We also reveal that, at the time of diabetes diagnosis, a worse clinical condition, characterized by higher glucose levels, lower fasting, and stimulated C-peptide, is related to higher EPC mobilization in peripheral blood. Taking into account that C-peptide has been shown to positively affect endothelial function by improving nitric oxide production and oxidative stress reduction [17], it is likely that its lowered levels may lead to adverse effects on the endothelium. These findings seem to confirm our hypothesis that increased recruitment of EPCs from bone marrow is a compensatory response to endothelial injury, and this suggests that EPC levels and the severity of endothelial damage are positively correlated. This may indicate that the endothelium of well-controlled T1D patients is less damaged, as reflected by the lower amount of EPCs in their peripheral blood, which could suggest a reduced risk of cardiovascular events in the future.

What is more, no statistically significant difference in CEC levels were observed between children with newly diagnosed T1D and their non-diabetic counterparts. However, their levels appear to be influenced by age and nutritional status. Positive correlations were observed between CEC levels and age, BMI at diagnosis, and BMI in subsequent years. It is known that obesity and a co-existing inflammatory state [24] contribute to the deterioration of endothelial function [25], which may be reflected by an increased number of CECs. Our findings also suggest that older age at the time of T1D diagnosis could be associated with a higher degree of endothelial dysfunction. However, considering the slight age difference between the control and study groups, further investigation is required to confirm this hypothesis.

We also noticed that higher levels of CECs were observed in patients who presented partial remission of the disease at 6 months after diagnosis. Although CECs are usually considered to be markers of vascular damage, we hypothesize that, in the context of partial remission, their elevation may also reflect an active endothelial repair process. Improved glycemic control during this phase [26] could promote vascular regeneration, with increased CEC levels suggesting enhanced endothelial turnover rather than progressive damage. This interpretation remains novel and speculative, yet provides a valuable direction for future investigations.

In addition to assessing EPC and CEC levels separately, we also investigated the relationship between their ratios and the patient’s clinical condition at the early stage of diabetes. EPC/CEC ratio is used to characterize the dynamic interaction between endothelial regeneration and injury [27]. In our study, patients with a more benign clinical course presented lower EPC/CEC ratios. Moreover, we found a positive correlation between EPC/CEC ratio and glucose level at hospital admission and a negative correlation with age, BMI, and pH and stimulated C-peptide levels. EPC/CEC ratios did not differ significantly between young individuals with T1D and healthy controls. A higher EPC/CEC ratio indicates the advantage of EPCs over CECs—a greater endothelial repair potential against vascular injury—whereas a lower ratio suggests the dominance of CECs—inadequate repair potential in the presence of sustained endothelial damage. However, as we mentioned earlier, higher EPCs levels may point to enhanced release of these cells from bone marrow in response to vascular damage; thus, in our study, a higher EPC/CEC ratio might reflect increased endothelial repair mechanisms in response to enhanced ongoing endothelial injury at the time of diabetes diagnosis. Hence, a higher EPC/CEC ratio, in parallel with an increased number of EPCs, may serve as a marker for increased endothelial damage at the time of disease onset. Regarding the correlation of EPC/CEC ratio with age and BMI, we hypothesize that chronic, long-term endothelial damage shifts the balance toward endothelial injury rather than repair, which associates with a significant increase in cardiovascular risk in people with higher bodyweight and longer-lasting diabetes. It should be highlighted that our interpretation of the EPC/CEC ratio remains hypothetical. Its biological significance in the pediatric T1D population has not yet been established, underlying the need for further investigations to clarify its potential clinical value.

To conclude, it is essential to take advantage of the preserved endothelial regenerative potential in children with newly diagnosed T1D by promoting EPC expansion to prevent cardiovascular complications in the future. A study of long-term T1D patients (the Medalists) found that, despite prolonged exposure to hyperglycemia, a subset of participants remained free of microvascular complications, which was associated with their maintained EPC levels [28]. These finding suggest that preserved EPC levels may be predictive of a lower risk for cardiovascular diseases and be associated with improved long-term outcomes in people with T1D [27]. Dietary interventions and lifestyle modifications have the potential to enhance the amount of EPCs in patients with diabetes and to reduce the risk of cardiovascular disease [8]. Although physical activity increases EPC levels in healthy individuals, this response is absent in individuals with T1D, which may be a consequence of a diminished ability for endothelial repair [29]. It is important to highlight that, according to some studies, combining metformin with insulin therapy in type 1 diabetes may positively influence EPC levels and their activity [30]. Moreover, when comparing EPC levels in young individuals with T1D using insulin pump therapy and multiple daily injections over a two-year observation period, increased EPC levels were observed in both groups; however, this rise was more pronounced in those receiving insulin pump therapy [31]. Additional studies support findings of elevated EPC levels in individuals treated with insulin pump therapy, with improved glycemic variability proposed as a potential contributing factor [6]. Similarly, ACE inhibition and statin therapy can positively affect EPC amounts and function in adults with diabetes [32].

### Limitations of This Study

Some limitations of this study need to be considered. It was conducted on a small group of subjects at a single research center. A larger sample size could provide greater power to detect small to moderate effects within the study group and to allow for stronger conclusions. The study did not include patients below 5 years of age, as these children usually present a more severe disease course and extremely short duration of disease symptoms; thus, we chose to maintain a more homogenous study group. Moreover, an age and BMI discrepancy was noted between the control and study groups. It is important to emphasize that most of our analyses focused on associations within the group of patients with T1D, and, therefore, comparisons between the healthy and diabetic cohorts should not affect our research objectives. However, this aspect requires further investigation in future research. Due to organizational and technical capabilities of our outpatient clinic, we used only fasting C-peptide levels to assess beta cell function during follow-up.

Unfortunately, no significant correlations between EPC and CEC levels at disease onset and the duration of partial remission in our study group were observed. Repeated measurement of EPC and CEC levels would provide more insight into the relation between studied cells and PR, and would make our results more reliable. Additionally, as we relied on descriptive trends, a deeper statistical approach with structural equation modeling would provide better interpretability for our conclusions.

## 5. Conclusions

In summary, our findings shed new light on the association between endothelial function and clinical condition at T1D diagnosis. We reveal a new potential for the application of EPCs and CECs as biomarkers reflecting both endothelial injury and reconstruction processes in children with T1D. This potential may serve as a foundation for further research aimed at the early detection of vascular complications and the assessment of therapeutic interventions targeted at endothelial protection. There is an urgent need for further research and the development of new therapies aimed at preserving endothelial repairing capacity in order to reduce cardiovascular risk in children with T1D.

## Figures and Tables

**Table 1:** Clinical condition scale at hospital admission. (DKA—diabetic ketoacidosis; diabetes symptoms: polydipsia, polyuria, nycturia, weight loss).

	Clinical Condition		
	Good	Moderate	Severe
**Diabetes symptoms**	Present	Present	Present
**Dehydration**	no/mild	Moderate	Marked
**pH (capillary)**	>7.3	7.2–7.3	<7.1
**Bicarbonate (mmol/L)**	>10	5–10	<5
**DKA**	No	mild/moderate DKA	severe DKA
**Consciousness**	Conscious	conscious/lethargic	unconscious

**Table 2:** Characteristics of the study group. Data presented as median and IQR. *p* < 0.05 are in bold. (DKA—diabetic ketoacidosis, DIR—daily insulin requirement, PR—partial remission, ns—non significant).

	Parameter	Type 1 Diabetes	Control Group	*p*-Value
	No.	45	20	
	Gender (% boys)	55.55%	52.38%	*p* = ns
	Age (yrs)	11 (8–12.5)	13.75 (10.25–15.25)	***p* = 0.0062**
**At onset**	Glucose level (mg/dL)	427 [317,495]	87 [82,93]	***p* < 0.00001**
	pH	7.35 (7.29–7.39)	-	-
	DKA (%)	31.11%	-	-
	BMI (kg/m^2^)	15.5 (14.0–17.9)	20.9 (18.25–23.25)	***p* = 0.000175**
	BMI-SDS	−0.53 (−1.32–0.37)	0.76 (−0.18–1.22)	***p* = 0.001766**
	HbA_1c_ (%)	12.32 (10.74–14.19)	5.07 (4.82–5.33)	***p* = 0.019**
	DIR (U/kg/24 h)	0.65 (0.5–0.77)	-	-
	PR (%)	24.44%	-	-
	Fasting C-peptide (ng/mL)	0.48 (0.31–0.65)	2.13 (1.67–2.59)	***p* = 0.019481**
	Stimulated C-peptide (ng/mL)	0.97 (0.74–1.53)	-	-
	No. of positive islet autoantibodies	1 (+)—21.5% 2 (+)—28.3% 3 (+)—50.2%	-	-
**3 months post diagnosis**	BMI (kg/m^2^)	18.05 (16.15–20.45)	-	-
	BMI-SDS	0.165 (−0.3–0.922)	-	-
	HbA_1c_ (%)	6.41 (5.97–6.92)	-	-
	DIR (U/kg/24 h)	0.45 (0.36–0.52)	-	-
	% of PR	53.85%	-	-
**6 months post diagnosis**	BMI (kg/m^2^)	18.35 (15.8–20.4)	-	-
	BMI-SDS	0.19 (−0.22–0.84)	-	-
	HbA_1c_ (%)	6.32 (5.95–6.71)	-	-
	DIR (U/kg/24 h)	0.465 (0.35–0.58)	-	-
	% of PR	51.35%	-	-
**12 months post diagnosis**	BMI (kg/m^2^)	18.28 (16.2–20.6)	-	-
	BMI-SDS	0.196 (−0.31–0.74)	-	-
	HbA_1c_ (%)	6.36 (5.87–6.86)	-	-
	DIR (U/kg/24 h)	0.555 (0.43–0.67)	-	-
	% of PR	33.33%	-	-
**24 months post diagnosis**	BMI (kg/m^2^)	19.05 (16.4–21.6)	-	-
	BMI-SDS	0.244 (−0.5–0.98)	-	-
	HbA_1c_ (%)	6.90 (6.45–7.38)	-	-
	DIR (U/kg/24 h)	0.73 (0.62–0.86)	-	-
	% of PR	14.71%	-	-
	C-peptide (ng/mL)	0.32 (0.13–0.8)	-	-

**Table 3:** χ^2^ analysis of selected clinical features and PR occurrence among T1D patients divided basing on EPC, CEC, and EPC/CEC ratio median levels. *p* values < 0.05 are in bold. (PR—partial remission, DKA—diabetic ketoacidosis, ns—non significant).

	EPC		CEC		EPC/CEC Ratio	
	>median	<median	>median	<median	>median	<median
**Glucose level** **at onset** **(>median)**	68.18%	34.78%	50.00%	52.17%	39.13%	63.64%
	***p* = 0.025**		ns		ns	
**DKA at onset**	31.82%	30.43%	22.73%	39.13%	43.48%	14.29%
	ns		Ns		***p* = 0.034**	
**C-peptide at** **onset within** **normal range**	26.32%	63.64%	50.00%	42.86%	59.09%	31.58%
	***p* = 0.017**		ns		ns	
**Stimulated C-peptide at onset** **(<median)**	72.22%	38.10%	45.00%	63.16%	45.45%	64.71%
	***p* = 0.033**		ns		Ns	
**PR at onset**	27.27%	21.74%	18.18%	30.43%	30.43%	18.18%
	ns		ns		Ns	
**PR 3 months** **post diagnosis**	50.00%	57.14%	61.11%	47.62%	57.89%	50.00%
	ns		ns		Ns	
**PR 6 months** **post diagnosis**	47.06%	55.00%	70.59%	35.00%	61.11%	42.11%
	ns		***p* = 0.030**		Ns	
**PR 12 months post diagnosis**	43.75%	25.00%	46.67%	23.81%	27.78%	38.89%
	ns		ns		Ns	
**PR 24 months post diagnosis**	21.43%	10.00%	13.33%	15.79%	10.53%	20.00%
	ns		ns		Ns	

**Table 4:** Correlations between studied cells (EPCs, CECs) and EPC/CEC ratios and the selected anthropometrical and laboratory parameters of T1D patients. Data presented as Spearman’s rank coefficient (ρ). *p* < 0.05 are in bold. (EPCs—endothelial progenitor cells, CECs—circulating endothelial cells, T1D—type 1 diabetes, BMI—body mass index).

	Age	BMI at Onset	BMI 12 Months Post Diagnosis	BMI 24 Months Post Diagnosis	Glucose at Onset	pH at Onset	Fasting C-Peptide at Onset	Stimulated C-Peptide at Onset
**EPC**	−0.08	0.01	0.22	0.21	0.17	−0.15	0.01	−0.09
**CEC**	**0.23**	**0.28**	**0.17**	0.16	−0.07	0.12	0.15	0.15
**EPC/CEC [ratio]**	−**0.27**	−**0.28**	−0.14	−0.19	**0.25**	**−0.32**	0.21	**−0.35**

## Data Availability

The raw data supporting the conclusions of this article will be made available by the authors on request.

## Abbreviations

The following abbreviations are used in this manuscript:

BMI	Body mass index
BMI-SDS	Body mass index standard deviation score
CECs	Circulating endothelial cells
CVD	Cardiovascular diseases
DIR	Daily insulin requirement
DMSO	Dimethyl sulfoxide
ECLIA	Electrochemiluminescence
EPCs	Endothelial progenitor cells
FBS	Fetal bovine serum
HbA1c	Glycated hemoglobin
HIF-1	Hypoxia-induced factor 1
IQR	Interquartile range
ISPAD	International Society of Pediatric and Adolescent Diabetes
PBMCs	Peripheral blood mononuclear cells
PBS	Phosphate-buffered saline
PR	Partial remission
SDF-1	Stromal-derived factor 1
T1D	Type 1 diabetes
